# Therapeutic Effects of Semaglutide on Nonalcoholic Fatty Liver Disease with Type 2 Diabetes Mellitus and Obesity: An Open-Label Controlled Trial

**DOI:** 10.3390/diseases12080186

**Published:** 2024-08-17

**Authors:** Ahmed I. Gad, Nevin F. Ibrahim, Noura Almadani, Rasha Mahfouz, Hanaa A. Nofal, Dina S. El-Rafey, Hossam Tharwat Ali, Amr T. EL-Hawary, Ayman M. E. M. Sadek

**Affiliations:** 1 Internal Medicine Department, Faculty of Medicine, Zagazig University, Zagazig 44519, Egypt; ahmedgadmed@yahoo.com (A.I.G.); nova.fayek@gmail.com (N.F.I.); dramrelhawary@yahoo.com (A.T.E.-H.); ayman.sadek@zu.edu.eg (A.M.E.M.S.); 2 Community and Psychiatric Mental Health Nursing Department, College of Nursing, Princess Nourah bint Abdulrahman University, P.O. Box 84428, Riyadh 11671, Saudi Arabia; naalmadani@pnu.edu.sa (N.A.); rmmahfouz@pnu.edu.sa (R.M.); 3 Community, Environmental Occupational Medicine Department, Faculty of Medicine, Zagazig University, Zagazig 44519, Egypt; hanofal@medicine.zu.edu.eg (H.A.N.); dselrafey@gmail.com (D.S.E.-R.); 4 Qena Faculty of Medicine, South Valley University, Qena 83621, Egypt

**Keywords:** semaglutide, nonalcoholic fatty liver disease, obesity, type 2 diabetes mellitus, GLP-1, clinical trial, diet study

## Abstract

Background: GLP-1 receptor agonists (GLP-1 RAs) have been shown to improve glycemic control and insulin sensitivity and reduce body weight in obese patients with type 2 diabetes mellitus (T2D). This trial sought to evaluate the therapeutic effect of oral and subcutaneous semaglutide in NAFLD and its sequelae in obesity and/or T2D. Methods: In an open-labelled intervention study, the sample was 180 patients classified into three parallel groups (1:1:1): group I received oral semaglutide, group II patients received injectable semaglutide, and group III received pioglitazone and/or vitamin E. Patients were evaluated at 6 and 12 months. Results: There was a substantial improvement in lipid profile, liver enzymes, and body mass index, especially in group II. As for HDL, only group II showed a consistent increase at both 6 months (51 ± 4.62 mg/dL) and 12 months (50.08 ± 2.45 mg/dL) compared with baseline (45.6 ± 6.37 mg/dL) (*p*-value < 0.001). Despite the non-significant difference in NAFLD fibrosis score (NFS) (*p*-value = 0.45 and 0.63), group II had significantly lower scores of the fibrosis-4 score (FIB-4), liver stiffness measurement (LSM), and controlled attenuation parameter (CAP) at 6 and 12 months (*p*-value < 0.001). **Conclusions:** Semaglutide improves lipid profile, liver steatosis, and fibrosis parameters and reduces the BMI in T2D and obese patients with NAFLD.

## 1. Introduction

There are marked changes in the contribution percentages of different etiologies for chronic liver disease (CLD), especially with the recent improvement in hepatitis C virus (HCV) point of care along with the progressively growing metabolic syndrome components linked to the new century lifestyle rules [1]. Non-alcoholic fatty liver disease (NAFLD) begins to rise as one of the major causes of CLD, with a worldwide prevalence of around 32%, with the highest prevalence (exceeding 40%) in the Americas and Southeast Asia [2,3].

NAFLD is defined as an increase in the liver fat content above 5%, composed mainly of triglycerides, which come from diet, or the lipolysis of adipose tissue due to insulin resistance that is further increased by steatosis in a bidirectional relationship [4]. Metabolic stress is characterized by insulin resistance and glucose and lipid metabolism disruption, leading to fatty acid build-up in the liver [5]. Liver triglyceride metabolism in turn leads to the release of reactive oxygen species and proinflammatory cytokines that recruit immune cells with subsequent non-alcoholic steatohepatitis (NASH) in about 30% of NAFLD. Subsequent activation of stellate cells induces fibrosis in about 20–40% of NASH; of them, 10–30% progress to cirrhosis. Multiple factors interchange in this sequence, including metabolic syndrome, oxidative stress, lifestyle, and genetic background [6,7,8].

Non-invasive diagnosis of NAFLD and NASH is a growing field that makes it a rapid, cheap, easy, safe, reproducible, and acceptable modality by using a combination of laboratory imaging techniques, including ultrasonography (US), computed tomography (CT), and magnetic resonance imaging (MRI), and fibrosis scores [7,8,9]. Except for the only approved unsustainable treatment modality for hepatic steatosis in the form of weight loss, no pharmaceutical agent until now has been approved to treat steatosis. Multiple drugs were suggested to decrease steatohepatitis without definitive treatment for the steatosis itself.

Glucagon-like peptide-1 (GLP-1) is an intestinal hormone that promotes insulin secretion and inhibits glucagon secretion from pancreatic islets. GLP-1 receptor agonists (GLP-1 RAs) agonists have a suggested multi-mechanistic role in the treatment of steatosis through each of the brain, adipose tissue, liver, pancreas, and gastrointestinal tract [10,11,12]. GLP-1 RAs have been shown to improve glycemic control and insulin sensitivity and reduce body weight [13]. A few running trials are focusing on the potential effects of GLP-1 RAs used for the treatment of type 2 Diabetes Mellitus (T2D) in the treatment of steatosis and improving the resulting fibrosis. This includes trials on the daily subcutaneous (SC) liraglutide and the newer, better-compliance semaglutide, either in once-weekly SC or oral forms [11,14,15]. Subcutaneous semaglutide, either once daily or once weekly, has been evaluated in a few placebo-controlled clinical trials that showed positive results, especially with liver steatosis and metabolic parameters [11,16,17,18]. However, there are no clinical trials that compare oral and subcutaneous semaglutide. Also, semaglutide was never compared with pioglitazone, which, along with GLP-1 Ras, is the preferred treatment for T2D with NAFLD, according to the recent American Diabetes Association (ADA) [19]. The objectives of the study were to determine the therapeutic effect of oral and SC semaglutide in NAFLD and its sequelae in T2D with obesity.

## 2. Methods

### 2.1. Study Design

The present study was an interventional, non-randomized, open-label controlled study conducted in the Internal Medicine Department at Zagazig University Hospitals, extending over one year, starting from study approval on 15 February 2023, until one-year follow-up of the last patients receiving medication (1 April 2024). Patients’ classification, data collection, and follow-up were performed by Dr. Ahmed I. Gad, the principal investigator, along with the help of the treating physicians. The study was registered on ClinicalTrials.gov, identified by Code No. ID: NCT05813249.

### 2.2. Sample Size and Technique

The sample size was based on the expected percent of resolution in NAFLD (36% versus 17% in semaglutide versus placebo), respectively [11], and the case:control ratio is 2:1. The sample size was 180 (60 in each group) at the confidence level 95% and power 80% using Open Epi program. The sample was classified according to the type of treatment into three groups by parallel allocation ratio 1:1:1 parallel in each group.

### 2.3. Study Population

**Figure 1:**
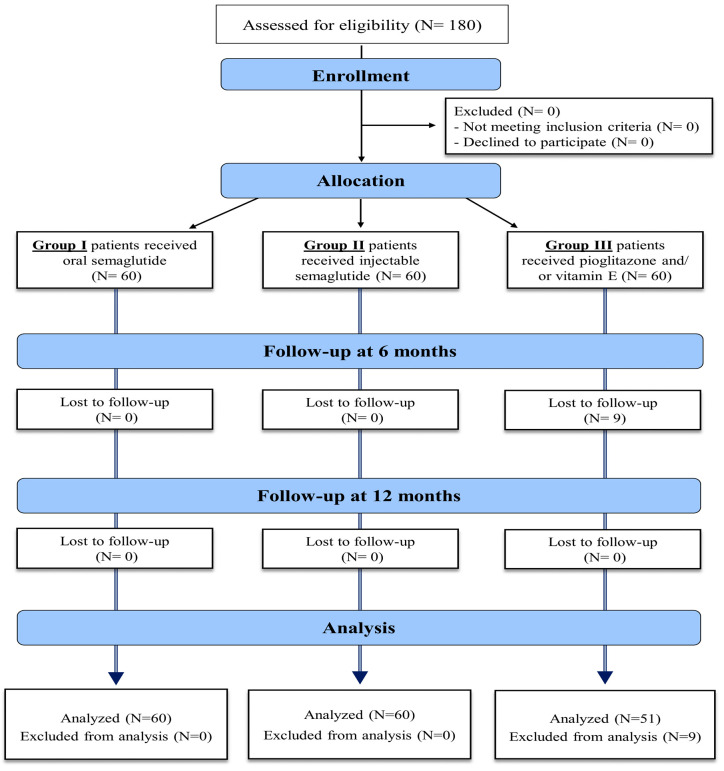
Enrollment process of the study patients.

The eligibility criteria of the study population included patients with the age of 18–75 years old, with type II DM, body mass index (BMI) > 30, diagnosed with NAFLD by non-invasive methods in the form of abdominal US or MRI, confirmed by a FibroScan with a controlled attenuation parameter (CAP), to evaluate hepatic steatosis and fibrosis severity. These assessments were conducted up to 21 weeks before the study began and repeated after treatment. Exclusion criteria included patients under 18 years of age, those with type 1 or insulin-dependent diabetes mellitus (DM), alcohol use, history of bariatric surgery, or those who tested positive for hepatitis C virus (HCV), hepatitis B virus (HBV), or human immunodeficiency virus (HIV). Additional exclusions were patients with peptic ulcer disease, secondary obesity due to hypothalamic or endocrine disorders, other causes of chronic liver disease (CLD), decompensated liver disease, a history of pancreatitis, hepato-biliary disorders, AST and ALT levels exceeding five times the upper normal limit, and severe cardiac disease. Patients who had received GLP-1 agonist treatment within 90 days prior to screening were also excluded. The enrollment process is detailed in Figure 1.

### 2.4. Study Interventions

Patients in group I received oral semaglutide (Rybelsus) with a starting dose of 3 mg daily and an up-titration to 14 mg for 48 weeks. Based on the PIONEER studies, oral semaglutide was approved by the Food and Drug Administration (FDA) in 2019 with a maximum dose of 14 mg daily [20,21]. Group II received SC injectable semaglutide (Ozempic) starting with 0.25 mg weekly for 4 weeks and up-titration gradually to reach 2 mg weekly for 48 weeks [11]. Group III received pioglitazone and/or vitamin E. According to the American Association for the Study of Liver Disease (AASLD), pioglitazone can improve liver steatosis, activity, and NAFLD resolution and can be considered in the context of patients with T2D, while vitamin E improves NAFLD in some patients [22,23,24,25]. The patients received medication at their own expense from a private pharmacy, which was done through revision of issued pharmacies by treating physicians.

### 2.5. Outcome Measures

Upon enrollment in the study, all patients underwent thorough medical history and complete clinical examination, including local examination of the liver, calculation of BMI, and measurement of waist circumference. Baseline laboratory investigations included complete blood count (CBC), liver function tests (AST, ALT, ALP, total and direct bilirubin, total protein, albumin, and INR), kidney function tests (creatinine and urea), lipid profiles (total cholesterol content, TGs, LDL, and HDL), fasting plasma glucose, and HbA1c. Liver stiffness measurement in kilopascals (kPa) representing the resistance of tissue to deformation and the controlled attenuation parameter (CAP) feature in the FibroScan measuring the attenuation of US waves as they pass through liver tissue [26] as a non-invasive assessment of steatosis were also measured. Furthermore, the fibrosis-4 score (FIB-4), which requires values of age, ALT, AST, and platelet count, was also measured, which requires values of age, BMI, platelet count, albumin, hyperglycemia, and ALT/AST ratio, in addition to the NAFLD fibrosis score (NFS), which is used to separate NAFLD patients with or without advanced fibrosis [27]. At 6-month and 12-month follow-up timepoints, changes in BMI, AST, ALT, lipid profile, liver stiffness measurement, CAP, FIB-4, and NFS were calculated for each group. Follow-up was done by treating physician and supervised by the principal investigator, Dr. Ahmed I. Gad.

### 2.6. Ethical Considerations

The study followed the ethical principles of the Declaration of Helsinki, and all experiments were performed in accordance with relevant regulations and guidelines. Participants were not exposed to any harm or unintended effects. The study was approved by the Institutional Research Review Board (IRB) of the Zagazig Faculty of Medicine (IRB#: 10461-15-2-2023). All patients consented to participate prior to the start of the trial.

### 2.7. Statistical Analysis

The data were collected and computerized, and the statistical analysis was performed using the Statistical Package for Social Science (SPS) program, version 27.0. Description of qualitative data was as frequencies and relative percentages, whereas quantitative data were expressed as mean ± standard deviation (SD) or median (range). The chi-square test, ANOVA with post hoc Tukey test, and Kruskal–Wallis with post hoc Dunn’s test were used to compare between groups according to data type. Repeated measure ANOVA test with post hoc Bonferroni test and Friedman test with post hoc Nemenyi test were used to compare different times in each group. *p*-value < 0.05 was considered significant, and <0.001 was deemed highly significant. Percentage of change was calculated as the percentage of difference between 12 months and baseline to the baseline values (12 months values—baseline values/baseline values).

## 3. Results

### 3.1. Baseline Characteristics of the Patients

In total, 180 patients were included in the study, of whom 94 (52.22%) were males and 58 (32.22%) were smokers, with an age range of 29 to 60 years and a mean (SD) of 47.33 (6.06) years. Most patients (69.44%) were without comorbidities. Only 48 (26.67%) patients were on oral hypoglycemic (OHG) drugs, while 27 (15%) were on insulin. Table 1 shows that there was no significant difference between the studied groups in all basic demographic, clinical, and laboratory data.

### 3.2. Changes in BMI, AST, and ALT among the Study Groups

The included participants had a mean (SD) BMI of 33.1 (2.12) kg/m^2^. Table 2 shows a statistically significant decrease in BMI of group I at both 6 months (31.02 ± 3.08) and 12 months (29.92 ± 3.15) compared with baseline (33.06 ± 2.1) (*p*-value < 0.001). Similarly, group II showed a trending decrease in BMI from 33.57 ± 2.17 at baseline to 29.65 ± 3.59 at 6 months and 28.25 ± 3.36 at 12 months (*p*-value < 0.001). Group III had a significant reduction at only 12 months (31.71 ± 3.43) compared with baseline (32.69 ± 2.04) (*p*-value = 0.03).

Regarding AST, only group II showed significant reduction at both 6 months (36 [20–105] U/L) and 12 months (34.5 [23–70] U/L) compared with baseline (38 [18–180] U/L) (*p*-value < 0.01 and <0.001, respectively). Similarly, concerning AST, only group II had significant changes across timepoints with values as follows: baseline (30.5 [18–137] U/L), 6 months (27 [18–68] U/L), and 12 months (26 [16–48] U/L) (*p*-value < 0.001 for both).

### 3.3. Changes in Lipid Profile among the Study Groups

Total cholesterol content was significantly decreased at 12 months compared with baseline for all groups: group I (238.06 ± 66.75 to 203.25 ± 22.68 mg/dL), group II (218.79 ± 38.22 to 182.08 ± 11.22 mg/dL), and group III (229.58 ± 48.56 to 197.1 ± 28.5 mg/dL) (*p*-values < 0.001 for all differences). Comparing results at 6 months to baseline values was significant in both groups I and II (*p*-value = 0.002, <0.001, respectively), while comparing values at 12 months to those at 6 months was only significant in group III with a *p*-value of 0.02.

As for triglycerides (TGs), values were significantly decreased at 12 months compared with baseline for all groups: group I (178.49 ± 75.53 to 125.07 ± 27.46 mg/dL), group II (172.96 ± 66.81 to 98.75 ± 24.48 mg/dL), and group III (181.98 ± 71.07 to 128.02 ± 44.41 mg/dL) (*p*-values < 0.001 for all differences). Regarding LDL, all groups had a significant reduction at 12 months compared with baseline as follows: group I (118.88 ± 41.06 to 102.1 ± 18.52 mg/dL; *p*-value = 0.007), group II (134.68 ± 32.63 to 97.67 ± 13.08 mg/dL; *p*-value < 0.001), and group III (121.5 ± 43.20 to 101.88 ± 21.78 mg/dL; *p*-value = 0.006). As for HDL, only group II showed a consistent increase at both 6 months (51 ± 4.62 mg/dL) and 12 months (50.08 ± 2.45 mg/dL) compared with baseline (45.6 ± 6.37 mg/dL) (*p*-value < 0.001 for both differences). Group I showed only a significant increase between 6 months (45.85 ± 3.86 mg/dL) and 12 months (48.02 ± 3.62 mg/dL) (*p*-value < 0.001). The details of the lipid profile are shown in Table 3.

### 3.4. Changes in Liver Stiffness and Fibrosis Parameters among the Study Groups

Table 4 shows the changes in NFS in the study groups at the follow-up timepoints. All groups showed significant reduction at 12 months compared with baseline: group I (−1.36 [−3.17 to 0.6] to −1.69 [−3.49 to 0.42]; *p*-value < 0.001), group II (−1.2 [−4.46 to 0.68] to −1.46 [−4.96 to 0.09]; *p*-value < 0.001), and group III (−1.49 [−3.54 to 0.89] to −1.71 [−4.06 to 1.08]; *p*-value < 0.001). Only group I showed a significant reduction at 6 months as well (−1.36 [−3.17 to 0.6] to −1.64 [−3.26 to 0.39]; *p*-value < 0.001), with an insignificant difference between values at 6 and 12 months (*p*-value = 0.51).

**Figure 2:**
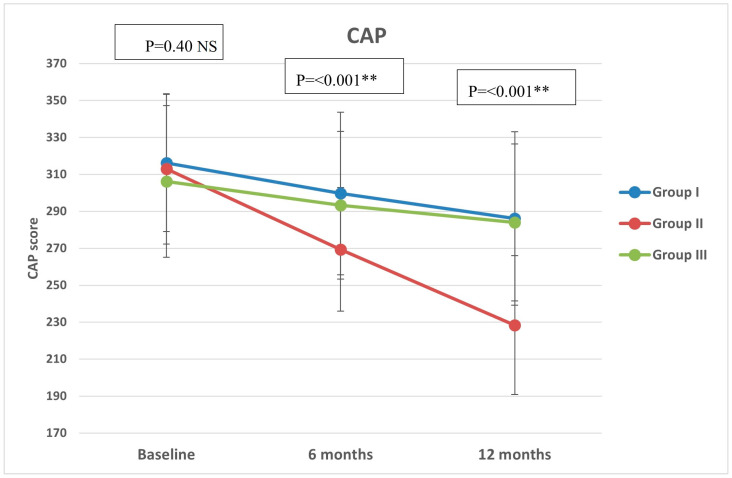
Controlled attenuation parameter (CAP) of the study groups through study timepoints. The *p*-value is for ANOVA test, NS: non-significant, ** highly significant *p*-value (<0.001).

**Figure 3:**
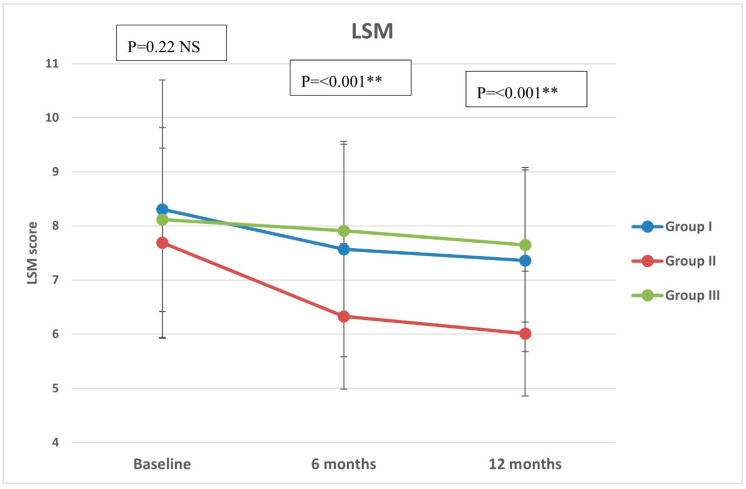
Liver stiffness measurement (LSM) of the study groups through study timepoints. The *p*-value is for ANOVA test, NS: non-significant, ** highly significant p-value (<0.001).

**Figure 4:**
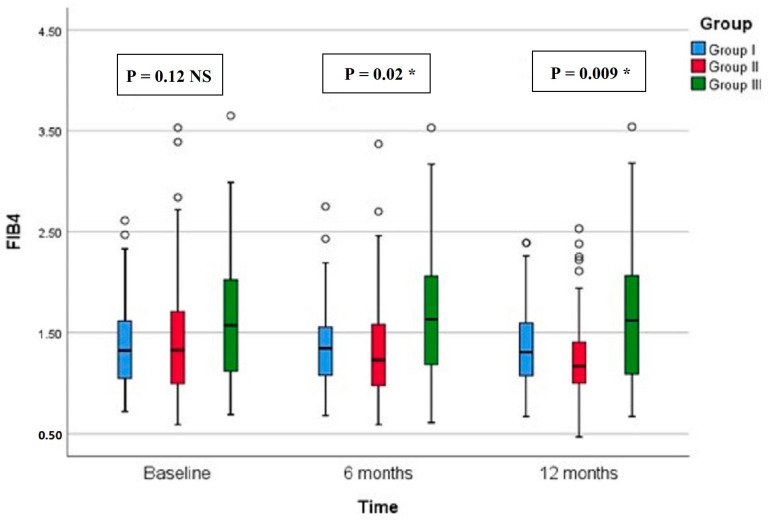
Fibrosis-4 score (FIB-4) of the study groups through study timepoints. The *p*-value is for Kruskal–Wallis test, NS: nob-significant, * significant *p*-value (<0.05).

Regarding CAP, there were significant differences in group II compared with groups I and III at both 6 months (*p* < 0.001, =0.005, respectively) and 12 months (*p* < 0.001 in both) (Figure 2). All groups showed significant reduction over timepoints (baseline, 6 months, and 12 months), with percentages of reduction of 8.72%, 26.74%, and 7.12%, respectively. Similarly, there were significant differences in LSM in group II compared with groups I and III at both 6 months (*p* < 0.001 in both) and 12 months (*p* < 0.001 in both) (Figure 3). All groups had a significant decrease in LSM at both 6 and 12 months compared with baseline, while only group II had a significant reduction at 12 months compared with 6 months. The percentages of reduction in groups I, II, and III were 10.06%, 20.34%, and 5.88%, respectively. Figure 4 shows significant differences in the FIB-4 score at 6 months (*p*-value = 0.02) and 12 months (*p*-value = 0.009), with insignificant differences at baseline (*p*-value = 0.12). Percentages of reduction were higher in group II as follows: FIB-4 (10.07% in group II versus 1.75% and 2% in groups I and III, respectively), CAP (26.74% in group II versus 8.72% in group I and 7.12% in group III), and LSM (20.34% versus 10.06% in group I and 5.88% in group III).

## 4. Discussion

Semaglutide, a GLP1-RA offered in subcutaneous and oral formulations, holds promise as a treatment for NAFLD due to its multifaceted mechanisms of action, rendering it a promising treatment for the disease [28]. Beyond its capacity for inducing weight loss, which correlates with histological improvements in NAFLD patients in a dose-dependent manner [29], semaglutide demonstrates additional liver benefits via anti-inflammatory and antioxidative pathways [30,31]. Moreover, research into GLP1-RAs has delved into their direct modulation of hepatic lipid metabolism, as evidenced by studies employing cell culture models on NAFLD [32]. Limited published studies have evaluated the effects of daily or weekly subcutaneous semaglutide in patients with NAFLD/NASH [17,33].

Most studies on GLP-1 RAs showed efficacy in reducing fat content and aminotransferases, with only a few studies focusing on histologic endpoints specifically with semaglutide and liraglutide [14,15]. Simaglutide is available in both once-weekly subcutaneous or daily tablets, and both are approved for T2D, while only subcutaneous at high doses is approved for obesity [34]. In this 12-month prospective trial, we evaluated the effect of once-weekly SC semaglutide (Ozempic), oral semaglutide (Rybelsus), and pioglitazone and/or vitamin E on NAFLD in patients with T2D. The present study included patients with a mean age of 47.33 years, which is younger than previous studies [35,36], with nearly half of them (49.43%) having moderate fatty liver infiltration with insignificant differences between study groups, which is a comparable percentage of moderate fatty liver infiltration of 44% reported by Volpe et al. [35]. GLP-1 RAs constitute a promising option for NAFLD with T2D since they can induce considerable weight loss, decrease visceral adipose tissue (VAT), and improve insulin sensitivity [8,37]. Most studies did not identify GLP-1 receptors in hepatocytes. So the changes in hepatic fat are believed to be mediated through changes in metabolic and weight-reduction actions [14,38,39]. A recent meta-analysis showed a correlation between a reduction in hepatic fat content and a reduction in BMI [40]. The mainstay of treatment for NAFLD is to achieve a weight reduction of at least 5–10% [36,41].

Our results confirm the reducing effect of semaglutide on BMI over the treatment period. Both forms of semaglutide induced a significant reduction at each follow-up timepoint, with the once-weekly SC injection having a greater reduction in BMI and percentage reduction at 12 months (16% vs. 9.44%). Our findings are in agreement with Volpe et al., who declared statistically and clinically relevant weight loss at each follow-up visit compared with baseline, with a mean loss in body weight of 10.3% at 12 months. Notably, our patients had a mean (SD) BMI of 33.1 (2.12) kg/m^2^, whereas in Volpe et al., it was 38.8 (8.3) kg/m^2^. Moreover, they reported an improvement in body composition with once-weekly semaglutide and, more importantly, VAT, which is the main contributor to insulin resistance and cardiovascular risks [35].

The initial analysis revealed no notable distinctions among the groups in terms of lipid profiles at the outset. Throughout the follow-up period, LDL levels remained insignificantly different across the groups, although they had a consistent and significant reduction at 6 and 12 months in the once-weekly injection group. Furthermore, noteworthy reductions in total cholesterol content and TGs levels, as well as significant elevations in HDL, were observed in the injection group compared with the other two groups at both the 6-month and 12-month timepoints. Conversely, we notice more improvement in all lipid profiles in the patients taking the injectable semaglutide, in agreement with Hachula et al. [42]. Also, numerous clinical trials confirm the beneficial effect of GLP-1 RAs on the lipid profile [36,43,44]. Low levels of HDL are a classic feature of NAFLD and are part of the disrupted lipid metabolism. No pharmacological agent has been effective in improving HDL levels sufficiently. We observed an improvement in HDL levels, which could be partially due to an enhanced intrahepatic synthesis of HDL, which, in turn, may affect NAFLD improvement. With confirmation from further studies, these data can contribute to a better comprehension of the cardiovascular beneficial effects of semaglutide in patients with T2D and NAFLD [13,35].

Generally, pharmacological treatment aims to achieve histological changes as well as clinical improvement [15]. Placebo-controlled trials of subcutaneous liraglutide and semaglutide showed the improving effect of GLP-1 RAs on liver histology in patients with NAFLD [45]. Our results indicated a statistically significant improvement in the injection group compared with the other groups in FIB-4, CAP, and LSM at 6 months and 12 months with higher percentages of reduction, but in regards to NFS, there was no significant difference between the three groups at three different times. Regarding NFS, there was a significant improvement in group I beginning at 6 months; on the other hand, in group II, significant improvement began at 12 months. Newsome et al. reported that in a specific phase II trial of once daily semaglutide (0.1, 0.2, and 0.4 mg) for 72 weeks in patients with NASH, liver steatosis and inflammation were reduced without exacerbating liver fibrosis, leading to resolution of NASH but without significant changes in liver fibrosis compared with placebo [11]. Similarly, satisfactory effects on liver steatosis but not liver stiffness were confirmed in a study using MRI to assess changes in liver parenchyma [17]. In addition, another study by Carretero-Gómez et al. reported that after 24 weeks of using subcutaneous semaglutide, significant reductions were observed in steatosis [36]. The LEAN study indicated that 39% of NASH patients receiving daily subcutaneous liraglutide (1.8 mg) for 48 weeks had biopsy-confirmed resolution of NASH, and the resolution rate was significantly higher than that in the placebo group (9%) [45].

### Strengths and Limitations

Our study has some strengths, including a robust study design, a well-standardized and comprehensive assessment of participants, a long-term follow-up (48 weeks), and the use of three lines of intervention: oral semaglutide, injectable semaglutide, and a control group (pioglitazone and/or vitamin E). Also, our study uses accurate diagnostic tools for point-of-care histological investigations in NAFLD, such as abdominal ultrasound, MRI, and FibroScan with CAP, to assess liver stiffness (kPa) and liver steatosis (dB/m). Nevertheless, the study does have some limitations. The first is the small sample size, although it is statistically adequate, which would not be sufficient for subgrouping (e.g., group III). The non-randomized nature of the trial introduces the possibility of confounders and potential bias. Furthermore, the absence of the study drug in hospitals or insurance institutions makes the patients acquire the drugs at their expense. This could be a source of selection bias, as only those who could afford or obtain the drug were eligible to enter the study. Notably, we lacked information on the diabetic duration. The study included patients with T2D and obesity, and hence, the results cannot be generalized to patients without obesity. The correlation between the imaging and laboratory parameters was not assessed. Despite these limitations, our trial confirms the beneficial results of previous studies with detailed laboratory and imaging assessments of NAFLD patients.

## 5. Conclusions

This 12-month interventional study proves the beneficial effects of semaglutide, either oral or subcutaneous, on body weight, insulin resistance, impaired liver function, lipid profile, and hepatic steatosis. Furthermore, significant improvement in radiological parameters of fibrosis in terms of FIB-4, CAP, and LSM was also observed, paving the way for more clinical involvement of semaglutide in NAFLD patients with T2D and obesity. Further randomized clinical trials of oral semaglutide with larger sample sizes, including NAFLD patients with and without diabetes, and with an active control group are warranted.

## Figures and Tables

**Table 1:** Baseline characteristics of the included patients among the study groups.

Variable		Group I (Rybelsus) (*n* = 60)		Group II (Ozempic) (*n* = 60)		Group III (Conventional) (*n* = 60)		Test	*p*
Age (years)	Mean ± SD	47.8 ± 5.41		46.83 ± 6.99		47.35 ± 5.74		0.38	0.69
	Range	35–58		29–60		37–59			NS ^#^
Variable		No	%	No	%	No	%	Test	*p*
Sex	Female	28	46.7	30	50	28	46.7	0.18	0.92
	Male	32	53.3	30	50	32	53.3		NS ^$^
Residence	Rural	31	51.7	32	53.3	31	51.7	0.05	0.98
	Urban	9	48.3	28	46.7	29	48.3		NS ^$^
Co-morbidities	No	42	70	36	60	47	78.3	7.41	0.49 NS ^$^
	HTN	13	21.7	19	31.7	12	20		
	IHD	1	1.7	1	1.7	1	1.7		
	Gout	3	5	3	5	0	0		
	Hypothyroidism	1	1.7	1	1.7	0	0		
Drugs	OHG	13	21.7	18	30	17	28.3	1.19	0.55 NS ^$^
	Insulin	6	10	7	11.7	4	6.7	0.91	0.63 NS ^$^
	ACEI	1	1.7	3	5	1	1.6	1.65	0.44 NS ^$^
	ARB + CCB	3	5	4	6.7	3	5	0.21	0.90 NS ^$^
	BB	0	0	1	1.7	0	0	2.01	0.37 NS ^$^
	Thiazide	0	0	1	1.7	0	0	2.01	0.37 NS ^$^
Smoking	No	40	66.7	44	73.3	38	63.3	1.43	0.49
	Yes	20	33.3	16	26.7	22	36.7		NS ^$^
PA U/S	Mild fatty	16	26.6	23	38.3	24	40	6.01	
	Moderate	31	51.7	32	53.4	26	43.3		0.20
	Severe	13	21.7	5	8.3	10	16.7		NS ^$^
FBG: (mg/dL)	Mean ± SD	110.22 ± 23.09		120.58 ± 32.32		113.65 ± 22.68		2.40	0.09 NS ^#^
HbA1c: (%)	Mean ± SD	7.6 ± 0.57		7.8 ± 0.87		7.41 ± 0.64		2.26	0.11 NS ^#^
WBCs: (×10^3^/mm^3^)	Mean ± SD	8.17 ± 2.09		8.13 ± 1.79		8.81 ± 1.5		2.65	0.07 NS ^#^
Hb: (g/dL)	Mean ± SD	12.13 ± 0.98		12.37 ± 1.37		12.32 ± 0.90		0.77	0.47 NS ^#^
Platelets: (×10^3^/mm^3^)	Mean ± SD	265.4 ± 53.39		269.53 ± 61.42		284.53 ± 49.33		2.02	0.14 NS ^#^
T. Bilirubin: (mg/dL)	Median (range)	0.7 (0.4–1.2)		0.72(0.4–2.3)		0.76(0.38–1.3)		1.06	0.59 NS ^^^
D. Bilirubin: (mg/dL)	Median (range)	0.16(0.08–0.7)		0.18(0.08–1.2)		0.19(0.08–0.68)		2.61	0.27 NS ^^^
Albumin: (g/dL)	Mean ± SD	4.13 ± 0.33		4.01 ± 0.35		4.02 ± 0.27		2.79	0.06 NS ^#^
Total protein: (g/dL)	Mean ± SD	7.35 ± 0.38		7.36 ± 0.37		7.47 ± 0.34		2.15	0.12 NS ^#^
ALP: (U/L)	Mean ± SD	100.83 ± 23.24		98.53 ± 18.77		106.55 ± 25.12		2	0.14 NS ^#^
INR:	Mean ± SD	0.98 ± 0.09		1 ± 0.09		1.02 ± 0.08		2.67	0.07 NS ^#^
Creatinine: (mg/dL)	Mean ± SD	0.73 ± 0.11		0.76 ± 0.15		0.75 ± 0.15		0.60	0.55 NS ^#^
Urea: (mg/dL)	Mean ± SD	13.49 ± 2.21		13.83 ± 2.24		13.95 ± 1.87		0.07	0.93 NS ^#^

Abbreviations: SD: standard deviation, NS: non-significant (*p* > 0.05), PA U/S: pelviabdominal ultrasound, FBG: fasting blood glucose, HbA1c: glycated hemoglobin, WBCs: white blood cells, Hb: hemoglobin, T. Bilirubin: total bilirubin, D. Bilirubin: direct bilirubin, ALP: alkaline phosphatase, INR: international randomized ratio. ^#^: ANOVA test (F), ^: Kruskal–Wallis test, ^$^: Chi square test (χ^2^).

**Table 2:** BMI and LFTs at different times of follow-up among the study groups.

Variable		Group I (Rybelsus) (*n* = 60)	Group II (Ozempic) (*n* = 60)	Group III (Conventional) (*n* = 51)	Test	*p*	Post hoc †
BMI: (kg/m^2^)	Baseline:	33.06 ± 2.1	33.57 ± 2.17	32.69 ± 2.04	2.48 ^#^	0.09 NS	-
	6 months	31.02 ± 3.08	29.65 ± 3.59	31.78 ± 2.89	6.28 ^#^	0.002 *	0.06 NS ^1^ 0.42 NS ^2^ 0.002 *^3^
	12 months	29.92 ± 3.15	28.25 ± 3.36	31.71 ± 3.43	15.08 ^#^	<0.001 **	0.02 *^1^ 0.43 NS ^2^ <0.001 **^3^
Post hoc Bonferroni		<0.001 **^a^ <0.001 **^b^ <0.001 **^c^	<0.001 **^a^ <0.001 **^b^ <0.001 **^c^	0.12 NS ^a^ 0.03 *^b^ 0.98 NS ^c^			
% of reduction		9.44%	16%	5.94%			
AST: (U/L)	Baseline:	45.5 (19–88)	38 (18–180)	50 (10–161)	0.59	0.75 NS	-
	6 months	45 (18–82)	36 (20–105)	48 (21–141)	17.5 ^	<0.001 **	0.004 *^1^ 0.99 NS ^2^ <0.001 **^3^
	12 months	43.5 (30–73)	34.5 (23–70)	44 (21–115)	29.3 ^	<0.001 **	<0.001 **^1^ 0.97 NS ^2^ <0.001 **^3^
Post hoc Nemenyi test		0.55 NS ^a^ 0.40 NS ^b^ 0.15 NS ^c^	0.01 *^a^ <0.001 **^b^ 0.18 NS ^c^	0.28 NS ^a^ 0.06 NS ^b^ 0.50 NS ^c^			
% of reduction		4.40%	17.64%	12%			
ALT: (U/L)	Baseline:	38 (18–137)	30.5 (18–137)	44 (20–116)	5.12 ^	0.08 NS	-
	6 months	36.5 (14–123)	27 (18–68)	43 (29–114)	50.8 ^	<0.001 **	<0.001 **^1^ 0.01 *^2^ <0.001 **^3^
	12 months	36 (22–59)	26 (16–48)	42 (27–114)	80.2 ^	<0.001 **	<0.001 **^1^ 0.001 *^2^ <0.001 **^3^
Post hoc Nemenyi test		0.20 NS ^a^ 0.052 NS ^b^ 0.18 NS ^c^	<0.001 **^a^ <0.001 **^b^ 0.23 NS ^c^	0.84 NS ^a^ 0.65 NS ^b^ 0.06 NS ^c^			
% of reduction		11.55%	27.62%	6.9%			

Abbreviations: BMI: body mass index, AST: aspartate aminotransferase, ALT: alanine aminotransferase. Data expressed as mean ± SD (standard deviation) or median (range). ^#^: ANOVA test (F), ^: Kruskal–Wallis test, NS: non-significant (*p* > 0.05), *: significant (*p* < 0.05), **: highly significant. (*p* < 0.001). ^†^ Post hoc: Tukey for ANOVA and Dunn’s test for KW. ^1^: group I versus group II, ^2^: group I versus group III, ^3^: group II versus group III. ^a^: baseline versus 6 m, ^b^: baseline versus 12 m, ^c^: 6 m versus 12 m.

**Table 3:** Lipid profile at different times of follow-up among the study groups.

Variable		Group I (Rybelsus) (*n* = 60)	Group II (Ozempic) (*n* = 60)	Group III (Conventional) (*n* = 51)	Test	*p*	Post hoc Tukey
Total cholesterol content: (mg/dL)	Baseline:	238.06 ± 66.75	218.79 ± 38.22	229.58 ± 48.56	2.01	0.14 NS ^#^	-
	6 months	208.3 ± 43.85	190.7 ± 18.34	214.75 ± 40.66	6.81	0.001 *^#^	0.02 *^1^ 0.61 NS ^2^ 0.002 *^3^
	12 months	203.25 ± 22.68	182.08 ± 11.22	197.1 ± 28.5	15.2	<0.001 **^#^	<0.001 **^1^ 0.30 NS ^2^ 0.001 *^3^
Post hoc Bonferroni		0.002 *^a^ <0.001 **^b^ 0.93 NS ^c^	<0.001 **^a^ <0.001 **^b^ <0.001 **^c^	0.30 NS ^a^ <0.001 **^b^ 0.02 *^c^			
% of reduction		8.02%	14.03%	10.73%			
TGs: (mg/dL)	Baseline:	178.49 ± 75.53	172.96 ± 66.81	181.98 ± 71.07	0.23	0.80 NS ^#^	-
	6 months	147.62 ± 44.44	124.42 ± 33.5	161.78 ± 57.76	9.59	<0.001 **^#^	0.02 *^1^ 0.24 NS ^2^ <0.001 **^3^
	12 months	125.07 ± 27.46	98.75 ± 24.48	128.02 ± 44.41	14.2	<0.001 **^#^	<0.001 **^1^ 0.88 NS ^2^ <0.001 **^3^
Post hoc Bonferroni		0.002 *^a^ <0.001 **^b^ <0.001 **^c^	<0.001 **^a^ <0.001 **^b^ <0.001 **^c^	0.35 NS ^a^ <0.001 **^b^ 0.03 *^c^			
% of reduction		24.59%	39.25%	14.86%			
LDL: (mg/dL)	Base line:	118.88 ± 41.06	134.68 ± 32.63	121.5 ± 43.20	2.79	0.06 NS ^#^	-
	6 months	110.38 ± 27.86	106.32 ± 18.98	107.22 ± 26.71	0.44	0.64 NS ^#^	-
	12 months	102.1 ± 18.52	97.67 ± 13.08	101.88 ± 21.78	1.14	0.32 NS ^#^	-
Post hoc Bonferroni		0.12 NS ^a^ 0.007 *^b^ 0.02 *^c^	<0.001 **^a^ <0.001 **^b^ <0.001 **^c^	0.06 NS ^a^ 0.006 *^b^ 0.54 NS ^c^			
% of reduction		20.83%	26.67%	16.54%			
HDL: (mg/dL)	Base line:	46.25 ± 5.45	45.6 ± 6.37	44.39 ± 5.18	1.48	0.23 NS ^#^	-
	6 months	45.85 ± 3.86	51 ± 4.62	46.22 ± 4.31	26.6	<0.001 **^#^	<0.001 **^1^ 0.90 NS ^2^ <0.001 **^3^
	12 months	48.02 ± 3.62	50.08 ± 2.45	46.67 ± 4.36	13.4	<0.001 **^#^	0.004 *^1^ 0.11 NS ^2^ <0.001 **^3^
Post hoc Bonferroni		0.99 NS ^a^ 0.12 NS ^b^ <0.001 **^c^	<0.001 **^a^ <0.001 **^b^ 0.50 NS ^c^	0.15 NS ^a^ 0.06 NS ^b^ 0.99 NS ^c^			
% of increase		5.39%	11.91%	6.62%			

Data expressed as mean ± SD (standard deviation) or median (range). #: ANOVA test (F), NS: non-significant (*p* > 0.05), *: significant (*p* < 0.05), **: highly significant. (*p* < 0.001). ^1^: group I versus group II, ^2^: group I versus group III, ^3^: group II versus group III. ^a^: Baseline versus 6 m, ^b^: baseline versus 12 m, ^c^: 6 m versus 12 m.

**Table 4:** NAFLD fibrosis score at different times of follow-up among the study groups.

Variable		Group I (Rybelsus) (*n* = 60)	Group II (Ozempic) (*n* = 60)	Group III (Conventional) (*n* = 51)	KW	*p*	Post hoc Dunn’s
NFS:	Baseline:	−1.36 (−3.17 to 0.6)	−1.2 (−4.46 to 0.68)	−1.49 (−3.54 to 0.89)	1.93	0.38	-
	6 months	−1.64 (−3.26 to 0.39)	−1.54 (−4.37 to 0.46)	−1.61 (−3.75 to 0.98)	1.62	0.45	-
	12 months	−1.69 (−3.49 to 0.42)	−1.46 (−4.96 to 0.09)	−1.71 (−4.06 to 1.08)	0.93	0.63	-
Post hoc Nemenyi test		<0.001 **^a^ <0.001 **^b^ 0.51 NS ^c^	0.70 NS ^a^ 0.003 *^b^ 0.13 NS ^c^	0.11 NS ^a^ 0.003 *^b^ 0.71 NS ^c^			
% of reduction		20.74%	13.73%	15.28%			

Data expressed as median (range). NFS: NAFLD fibrosis score, KW: Kruskal–Wallis test, NS: non-significant (*p* > 0.05), *: significant (*p* < 0.05), **: highly significant. (*p* < 0.001 ^a^: baseline versus 6 m, ^b^: baseline versus 12 m, ^c^: 6 m versus 12 m.

## Data Availability

The datasets generated during and/or analyzed during the current study are available from the corresponding author upon reasonable request.
